# Synthesis of Silver-Impregnated Magnetite Mesoporous Silica Composites for Removing Iodide in Aqueous Solution

**DOI:** 10.3390/toxics9080175

**Published:** 2021-07-26

**Authors:** Sang-Eun Jo, Jung-Weon Choi, Sang-June Choi

**Affiliations:** 1 School of Architectural, Civil, Environmental, and Energy Engineering, Kyungpook National University, 80 Daehak-ro, Buk-gu, Daegu 41566, Korea; jse0722@knu.ac.kr; 2 Green Carbon Catalysis Center, Korea Research Institute of Chemical Technology, 141 Gajeong-ro, Yuseong, Daejeon 34114, Korea; jwchoi92@krict.re.kr

**Keywords:** iodine, radionuclide, radioactive liquid waste, mesoporous silica, adsorption

## Abstract

Mag@silica-Ag composite has a high sorption ability for I^−^ in aqueous solution due to its high surface area and strong affinity for the studied anion. The material adsorbed I^−^ rapidly during the initial contact time (in 45 min, η = 80%) and reached adsorption equilibrium after 2 h. Moreover, mag@silica-Ag proved to selectively remove I^−^ from a mixture of Cl^−^, NO_3_^−^ and I^−^. The adsorption behavior fitted the Langmuir isotherm perfectly and the pseudo-second-order kinetic model. Based on the Langmuir isotherm, the maximum adsorption capacity of mag@silica-Ag was 0.82 mmol/g, which is significantly higher than previously developed adsorbents. This study introduces a practical application of a high-capacity adsorbent in removing radioactive I^−^ from wastewaters.

## 1. Introduction

Iodine, with only one stable isotope, is the rate limiting substance in the production of thyroid hormones. It cannot be naturally produced by the human body, so it must be obtained from food sources, such as fish, eggs, nuts, meat, and seaweed. However, excessive accumulation of a radioactive iodine in the body leads to thyroid cancer, and iodine poisoning causing mouth, throat, and stomach burns [1]. From all 36 radioactive isotopes, iodine-131 is a major concern in any kind of radiation emissions released from a nuclear accident because it is volatile and highly radioactive, having a half-life of 8 days. As it is a beta and gamma emitter, it is highly carcinogenic. Iodine-131 can be easily absorbed by plants, where its concentration can increase tenfold or even more compared to the ground contamination [2]. The radioisotope is of further concern for the human body, as the thyroid gland has a maximum uptake of iodine [3]. Therefore, studies focusing on the removal of iodine from wastewaters are of crucial importance in order to prevent exposure to radioactive iodine.

The movement of iodine radionuclides through the environment is complex, as it can rapidly disperse. When reaching a water source, radioactive iodine changes into various forms such as iodide, iodate, or organic iodide, impeding the water disinfection process [4].

There are various methods for adsorbing and removing I^−^ in aqueous solutions. For example, inorganic anion exchangers are used to adsorb chloride or iodide anions but some of them are considered toxic and cannot be used to purify drinking water. Hence, metal oxides and metal hydroxides play a crucial role in the adsorption of anions in underwater systems. Several studies have demonstrated that iodine is successfully adsorbed by iron, aluminum oxide, cuprous oxide, soil, minerals, silver-impregnated activated carbon, and quartz under acidic conditions [5,6,7].

Nowadays, silica-based adsorbents are key materials for a plethora of applications due to the unique properties of silica: high porosity, large inner surface area, and high adsorption properties [8]. For example, I^−^ can be selectively adsorbed by impregnating a silica structure with silver. The formation of silver iodide species (AgI) after adsorption has already been reported in various studies [9,10,11,12,13,14,15,16].

In this context, the present study focuses on the removal of iodide ions from aqueous solutions using silver-impregnated silica-based adsorbents. Besides the adsorbent efficiency, a good material should be easily recovered from the aqueous media. Thus, magnetite was attached to the silica-based adsorbent. Magnetite is biodegradable, biocompatible, non-toxic, easy to synthesize, renewable, and readily isolated from solutions by applying an external magnetic field [17,18,19,20,21,22]. The obtained material, denoted as mag@silica-Ag, presented an adsorption capacity of 0.82 mmol/g, significantly higher than previously developed adsorbents.

## 2. Materials and Methods

### 2.1. Materials

All the solutions were prepared using deionized (DI) water (18.3 MΩ cm) obtained using a Barnstead E-pure Water Purification System (D4641, Barnstead, NH, USA). Iron(III) chloride hexahydrate (FeCl_3_·6H_2_O, 97%, M.W = 270.29 g/mol), iron(II) sulfate (FeSO_4_·7H_2_O, 98.0%, M.W = 278.02 g/mol), ethyl alcohol (C_2_H_5_OH, 94 vol.%, M.W = 46.07 g/mol), ammonia (NH_4_OH, 30%, M.W = 35.04 g/mol), 1 N-hydrochloric acid (1 M) (HCl, M.W = 36.5 g/mol), sodium chloride (NaCl, 99%, M.W = 58.44 g/mol), and sodium nitrate (NaNO_3_, 99%, M.W = 85.00 g/mol) were supplied by Duksan Chemicals Co., Yongin, Korea. Tetraethyl orthosilicate (TEOS, C_8_H_20_O_4_Si, 98%, M.W = 208.33 g/mol), (3-(2-Aminoethylamino)propyl) triethoxysilane (AAPTMS, C_8_H_22_N_2_O_3_Si, 80%), and hexadecyltrimethyl ammonium bromide (CTAB, C_19_H_42_BrN, 99%) were purchased from Sigma-Aldrich Chemical Co. (Milwaukee, WI, USA). Silver nitrate (AgNO_3_, 99.8%, M.W = 169.87 g/mol) was purchased from Daejung Chemicals Co., Daejeon, Korea. Potassium iodide standard solution (1000 ppm, KI) used for the calibration curves was supplied by Accu Standard Co., New Haven, CT, USA.

### 2.2. Preparation of Silver Functionalized Magnetic Silica Adsorbents

#### 2.2.1. Synthesis of Mag@silica Composites

In order to synthesis magnetite, 100 mL of 0.8 M FeCl_3_·6H_2_O was added to the same volume of 0.4 M FeSO_4_·7H_2_O, keeping the molar concentration ratio of Fe ions to 2:1. The precipitation was carried out at 298 K under vigorous stirring using aqueous NH_3_ solution. The precipitate was heated for 30 min at 353 K in an N_2_ atmosphere, washed with DI water several times, and dried in an oven at 333 K for a day [22].

The mesoporous silica-coated magnetite nanoparticles (mag@silica) were synthesized using the following method. The magnetite nanoparticles (1.5 g) were dispersed in 200 mL of ethanol using ultrasounds for 30 min. Subsequently, 4.5 g of CTAB, 1200 mL of DI water, 1.5 g of ammonia solution, and 450 mL of ethanol were added to the dispersed solution. The mixture was subjected to ultrasound treatment for another 15 min. The silica precursors (6.45 mL of TEOS) and AAPTMS (3.9 mL) were added to the solution under sonication for 2 h. The product was separated by centrifugation and washed several times with ethanol and DI water. The sorbent in the 0.003 N HCl/ethanol was sonicated for 30 min to remove the surfactant from the pores of the silica structure. The resulting precipitate was separated by centrifugation and washed several times with DI water.

#### 2.2.2. Synthesis of Mag@silica-Ag Composites

**Figure 1:**
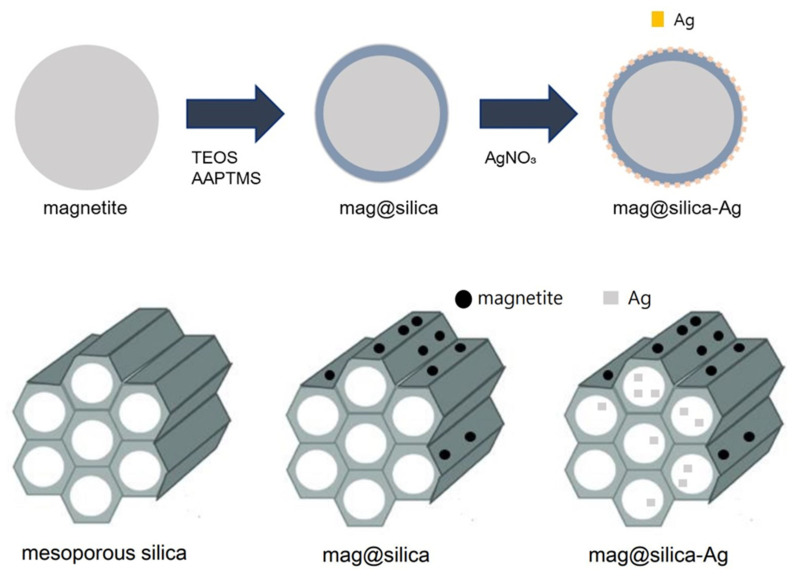
Synthesis of mag@silica-Ag.

The silver-impregnated magnetite-silica (mag@silica-Ag) was synthesized as follows: firstly, the mag@silica (1 g) was stirred in 100 mL of silver nitrate (0.1 M) until the pH increased to above 9.5; the solution was stirred at 323 K for 20 h using a shaking incubator (250 rpm). The product was separated by centrifugation and washed three times with DI water. Finally, the resulting precipitate was dried in an oven at 333 K for 12 h. The overall procedure is depicted in Figure 1.

### 2.3. Characterization

The surface morphology was studied using scanning electron microscopy (SEM, SU8220, Hitachi, Tokyo, Japan) and elemental analysis was determined using SEM-EDS (SU8220, Hitachi). The particle size was estimated using SEM. The specific surface area was determined by N_2_ adsorption (BET, Quadrasorb evo, Quantachrome, Boynton Beach, FL, USA). The pore size distribution was obtained using the Barret–Joynerv–Halenda (BJH) method. The functional groups present on the surface of the adsorbent were analyzed using Fourier-transform infrared spectroscopy (FT-IR, Frontier, PerkinElmer, Waltham, MA, USA). X-Ray photoelectron spectroscopy (XPS, NEXSA, ThermoFisher, Waltham, MA, USA) was used for surface composition analysis. The mag@silica and mag@silica-Ag were characterized by powder X-ray diffraction (XRD, EMPYREAN, Panalytical, Malvern, Worcestershire, UK). Using vibrating sample magnetometers (VSM, 7407-S, LakeShore, Carson, CA, USA), the magnetic properties of mag@silica and mag@silica-Ag were measured. Thermogravimetric analysis (TGA, Discovery SDT 650, TA Instruments, New Castle, DE, USA) was used to measure the thermal stability of the hybrid material. TGA analysis monitored and recorded the mass change over a temperature range of 30–700 °C at a heating rate of 10 °C min^−1^ under nitrogen flow.

### 2.4. Adsorption Experiments

#### 2.4.1. Adsorption Isotherms

**Figure 2:**
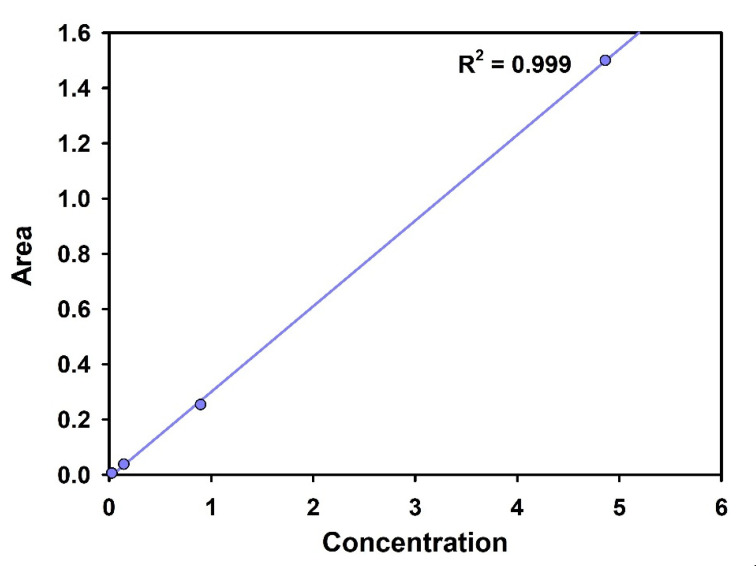
Standard calibration curve of I^−^.

As non-radioactive iodine behaves similar to the radioactive one, the adsorption experiments were conducted with non-radioactive I^−^. The I^−^ adsorption isotherm was determined by measuring the concentration of I^−^ left in the solution after the equilibrium was reached. The experiments were carried out for 24 h, with a 10-mL NaI solution having an I^−^ concentration in the range of 1–200 ppm. Standard KI solution was used to prepare the calibration curve (Figure 2). The concentrations before and after adsorption were determined using IC (Ion Chromatography, ICS-5000, Dionex, Sunnyvale, CA, USA).

Equilibrium adsorption capacity (*q_e_*) (mg/g) and removal efficiency (*R_I_^−^*) were calculated using the following equation:

(1) $$q_{e}=\left(C_{o}-C_{e}\right)\times \frac{V}{m}$$

(2) $$R_{I^{-}}=\frac{\left(C_{o}-C_{e}\right)}{C_{o}}\times 100$$

where, *C_o_* and *C_e_* indicate the initial and equilibrium concentrations of I^−^ in the aqueous solution, respectively. *V* (mL) is the volume of I^−^ solution, *m* (g) is the weight of the adsorbent, and $R_{I^{-}}$ is the removal efficiency.

The Langmuir adsorption model explains adsorption by assuming that the adsorbate behaves as an ideal gas at isothermal conditions. The Langmuir isotherm accounts for the surface coverage by balancing the relative rates of adsorption and desorption [23]. The Langmuir equation can be written as follows (Equation (3)):

(3) $$q_{e}=\frac{q_{m}bC_{e}}{1+bC_{e}}$$

where *C_e_* (mmol/L) is the concentration of adsorbate at equilibrium, *q_e_* (mmol/g) is the amount of adsorbate per gram of adsorbent, $q_{m}$ (mmol/g) is the maximum sorption capacity, and *b* (L/mmol) is the Langmuir constant related to adsorption capacity.

The performance of the adsorbents can be compared by applying the Freundlich equation, an empirical model widely used in environmental chemistry:

(4) $$q_{e}=K_{F}C_{e}{}^{N}$$

where *q_e_* (mmol/g) is the amount of adsorbate per gram of adsorbent, *C_e_* (mmol/L) is the equilibrium concentration of the solute in an aqueous solution, and $K_{F}$ and *N* are Freundlich constants. $K_{F}$ represents the adsorption capacity of the solid phase at a specific concentration of a solute in an aqueous solution, and the index *N* represents the magnitude and non-uniformity of the energy during the adsorption process. When *N* < 1, adsorption occurs at the adsorption site with high energy, and then adsorption occurs at the adsorption site with low energy. When *N* > 1, the solute already adsorbed on the solid phase changes the surface of the solid phase, increasing the amount of solute adsorption. When *N* = 1, it represents a solute distribution phenomenon.

#### 2.4.2. Adsorption Kinetics

The kinetic studies were done by placing 0.01 g of mag@silica-Ag adsorbent in 10 mL of 50 ppm I^−^ aqueous solution and stirring (250 rpm, 298 K) for 1, 2, 5, 10, 20, 45, and 60 min. The I^−^ concentration was determined using IC analysis, as mentioned in the previous section.

Two models were applied to analyze the kinetic data: a pseudo-first-order kinetic model (PFOKM) and a pseudo-second-order kinetic model (PSOKM) (Equations (5) and (6), respectively):

(5) $$\ln(q_{e}-q_{t})=\ln q_{e}-k_{1}t$$

(6) $$\frac{t}{q_{t}}=\frac{1}{k_{2}q_{e}{}^{2}}+\frac{1}{q_{e}}t$$

where $q_{e}$ and $q_{t}$ are the amounts of adsorbed I^−^ (mg/g) at equilibrium and *t*, the time, respectively; $k_{1}$ is the pseudo-first-order reaction rate constant (1/min), and $k_{2}$ is the pseudo-second-order reaction rate constant (g/mg∙min) [24].

#### 2.4.3. Effect of pH

To study the effect of pH upon the adsorption, 0.01 g of mag@silica-Ag were introduced into a 10 mL of I^−^ aqueous solution with a concentration of 50 ppm, stirred at 250 rpm, 298 K for 24 h. The pH was maintained between 1 and 11 using either HNO_3_ (0.1 M) or NaOH (0.1 M). The concentration of I^−^ was determined as previously mentioned.

#### 2.4.4. Effect of Co-Existing Ions

The effect of co-existing anions (Cl^−^ and NO_3_^−^) on iodide sorption onto the mag@silica-Ag composites was investigated. The initial concentration of I^−^ was maintained constant at 100 ppm, while the concentrations of the co-existing ions was varied: 10, 50, 100, and 200 ppm. In a typical reaction, 0.01 g of mag@silica-Ag was introduced into the solution containing just the I^−^ and stirred at 250 rpm, at 298 K for 24 h. The material was separated and reintroduced into solutions containing the co-existing ions, under the same conditions as above. The I^−^ concentration was determined as already mentioned. The distribution coefficient, *K_d_* (mL/g) of I^−^ at different concentrations of NaCl and NaNO_3_ was determined using the following equation:

(7) $$K_{d}=\frac{C_{o}-C_{e}}{C_{e}}\times \frac{V}{m}$$

where *C_o_* and *C_e_* are the initial and equilibrium concentrations (mg/g) of I^−^ in each solution, *V* the I^−^ solution volume (L), and *m* the mass of sorbent (g). Several factors such as the sorbent to solution ratio (*V/m*), initial concentration of the metal solution, solution composition, and the preparation method of materials must be considered to compare *K_d_* values accurately.

## 3. Results and Discussion

### 3.1. Characterization of the Adsorbent

**Figure 3:**
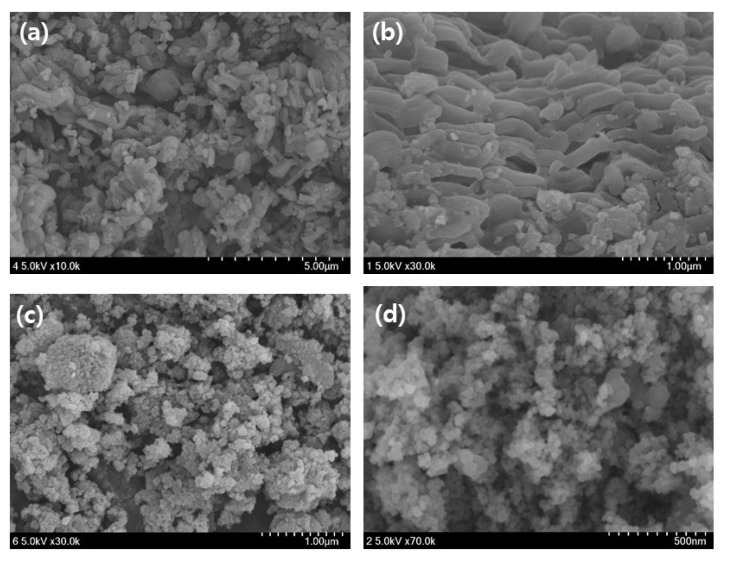

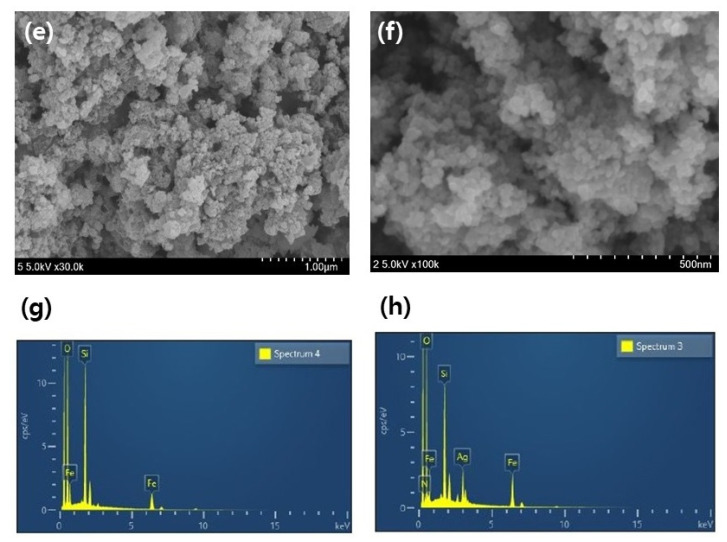
SEM image of (**a**,**b**) silica, (**c**,**d**) mag@silica, (**e**,**f**) mag@silica-Ag, and (**g**,**h**) EDS results of mag@silica and mag@silica-Ag.

The SEM images of the mag@silica-Ag, mag@silica, and silica are shown in Figure 3. A scanning electron microscopy (SEM) is useful for characterizing the morphological structure and size of magnetic nanoparticles. The diameter of the silica-based resin was estimated to be about 75–150 µm [25]. The shape of the conventional silica and the composite adsorbent in which silica is impregnated with magnetite and silver appeared completely different. Although there is no striking difference between mag@silica-Ag and mag@silica, the shape of mag@silica-Ag is more clustered than the parent material. Table 1 presents the results obtained from EDS analysis for the mag@silica-Ag sample. As expected, oxygen is present in the highest concentration followed by Fe, Si, N, and Ag. The presence of nitrogen is due to the precursors, CTAB and AAPTMS, used in the synthesis of silica.

**Figure 4:**
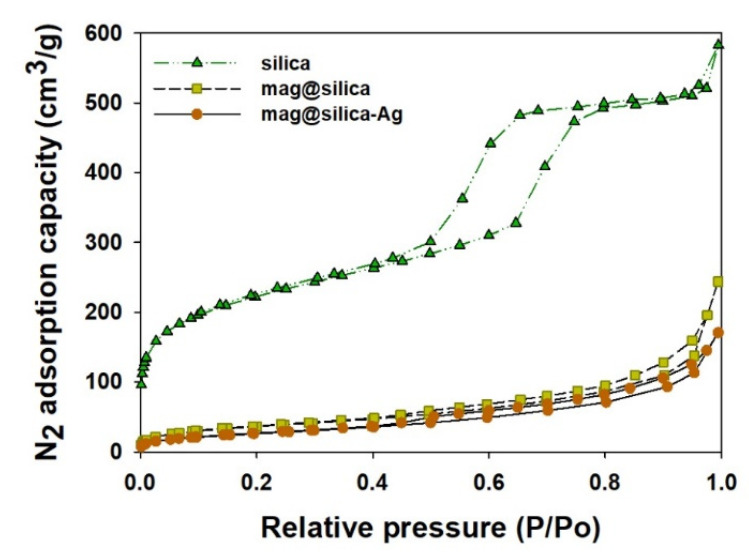
N_2_ adsorption-desorption isotherm at 77.3 K on silica, mag@silica, and mag@silica-Ag composites.

**Figure 5:**
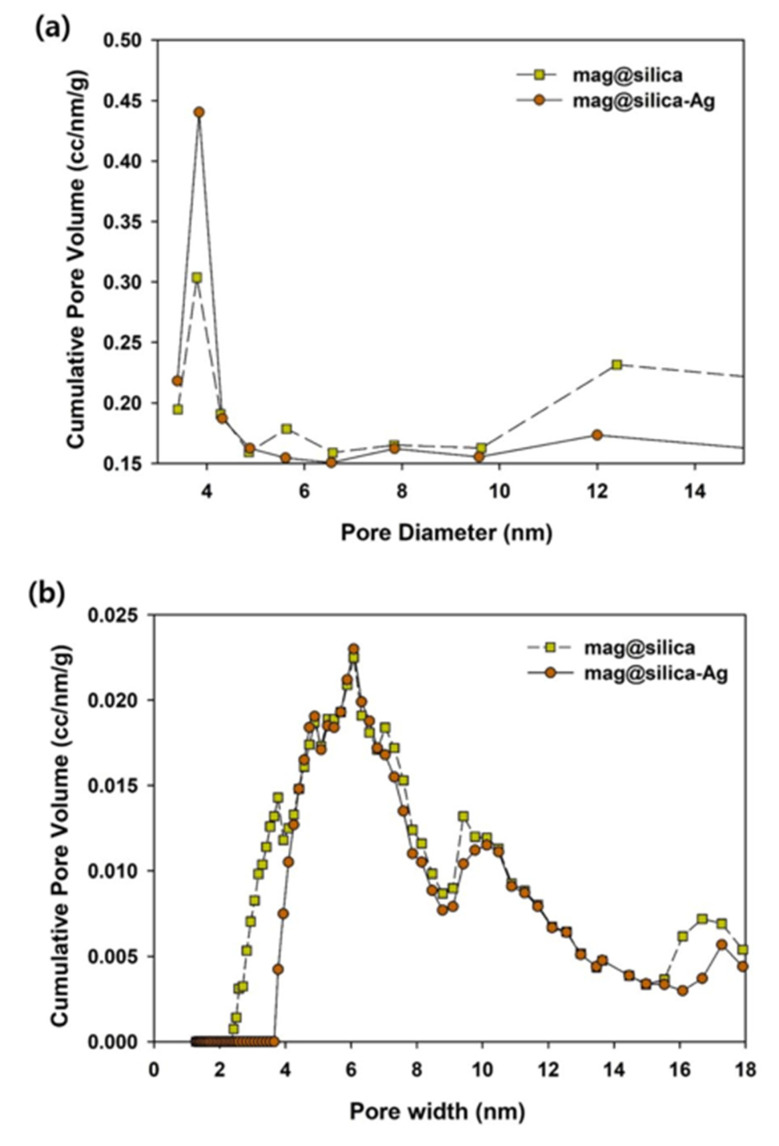
(**a**) Barrett–Joyner–Halenda (BJH) pore size distribution curves for the mag@silica and mag@silica-Ag, (**b**) the density functional theory (DFT) pore size distribution curves for the mag@silica and mag@silica-Ag.

The textural characterization of silica, mag@silica, and mag@silica-Ag composites was determined by BET surface area, BJH pore volume and size analysis, and density functional theory (DFT) curve (Figure 4 and Figure 5 and Table 2). The samples were pre-treated at a temperature of 423 K for an hour to remove any water present in the pores [26]. The BET surface area of silica was found to be 768.127 m^2^/g, whereas that of the mag@silica composite was 128.133 m^2^/g and mag@silica-Ag was 96.570 m^2^/g, respectively. The adsorption-desorption isotherms (Figure 4) show hysteresis at high relative pressure values where large pores are present. This type of hysteresis is specific to the macroporous-mesoporous materials as the nanocomposite used in this study. The BJH model applied to the desorption branch of the isotherm indicates that the powder is macroporous and that the synthesis of mag@silica-Ag occurs with the reduction of the pore’s width [27]. According to the IUPAC guidelines regarding the hysteresis, the adsorbent developed in this study has a macropore N_2_ adsorption structure [28,29]. Neither of the two materials adsorbed in the micro and mesopores. As N_2_ adsorption and desorption are influences by the pore structures, it can be safely concluded that mag@silica-Ag has a macropore structure [30]. According to Figure 5a, the silver-impregnated composite presents more smaller pores than the parent material.

As listed in Table 2, silica has a larger surface area, pore size, and pore volume than mag@silica and mag@silica-Ag. The large surface area and the large pores are essential in increasing the adsorption capacity of the adsorbents. Although the surface areas of mag@silica and mag@silica-Ag decreased, their value is sufficiently large compared to any other adsorbents [3,22,27,28,31]. Because the pore volume of mag@silica-Ag was lower than the bare silica, it can be concluded that the silver was deposited inside the pores of the material [32].

**Figure 6:**
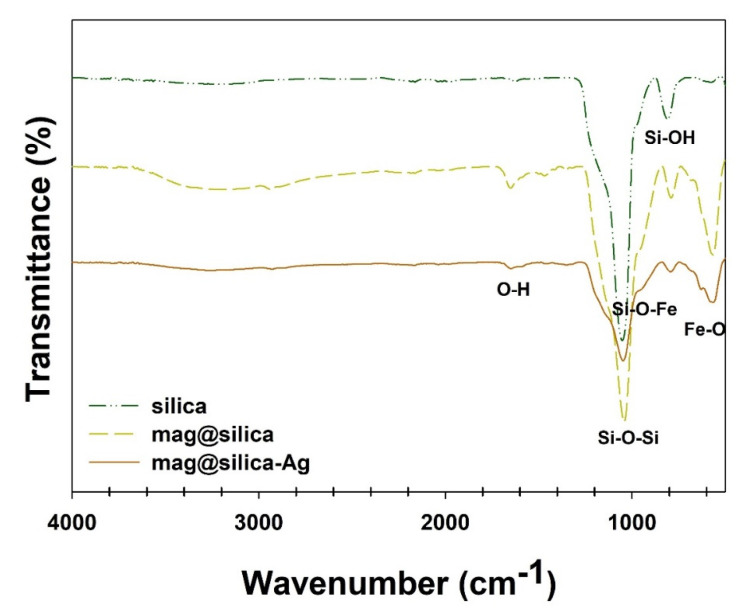
FT-IR spectra of silica, mag@silica, and mag@silica-Ag composites.

Figure 6 shows the FT-IR spectra of the silica, mag@silica, and mag@silica-Ag. For the silica, mag@silica, and mag@silica-Ag samples, the vibrational bands at ~567 cm^−1^ originate from the *v* (Fe-O) lattice vibrations. The mag@silica presents bands at 1049 cm^−1^, 815 cm^−1^, and 966 cm^−1^ corresponding to the stretching vibrations of *v* (Si-O-Si), *v* (Si-OH), and *v* (Si-O-Fe), respectively. The vibrational bands at ~580 cm^−1^ observed for mag@silica and mag@silica-Ag are due to the *ν* (Fe-O) lattice vibrations [22,33,34].

**Figure 7:**
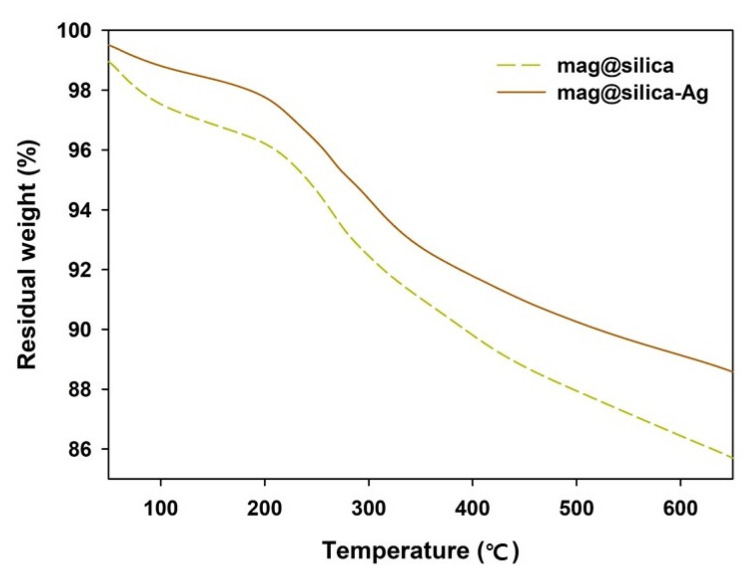
TGA curves of mag@silica and mag@silica-Ag composites.

The TGA curves of the mag@silica and the mag@silica-Ag are shown in Figure 7. Mag@silica presents a total weight loss of 11%, while the mag@silica-Ag a total loss of 15%. In both cases, the weight loss takes place in two steps, prior to 600 °C: (i) below 210 °C, due to the loss of water in the sample and (ii) between 210 and 550 °C due to the thermal decomposition of the organic species found in the materials. The TGA curve above 600 °C indicates that the organic species of the magnetic composite nanoparticles are completely decomposed and the Fe_3_O_4_ nanoparticles remain [35].

**Figure 8:**
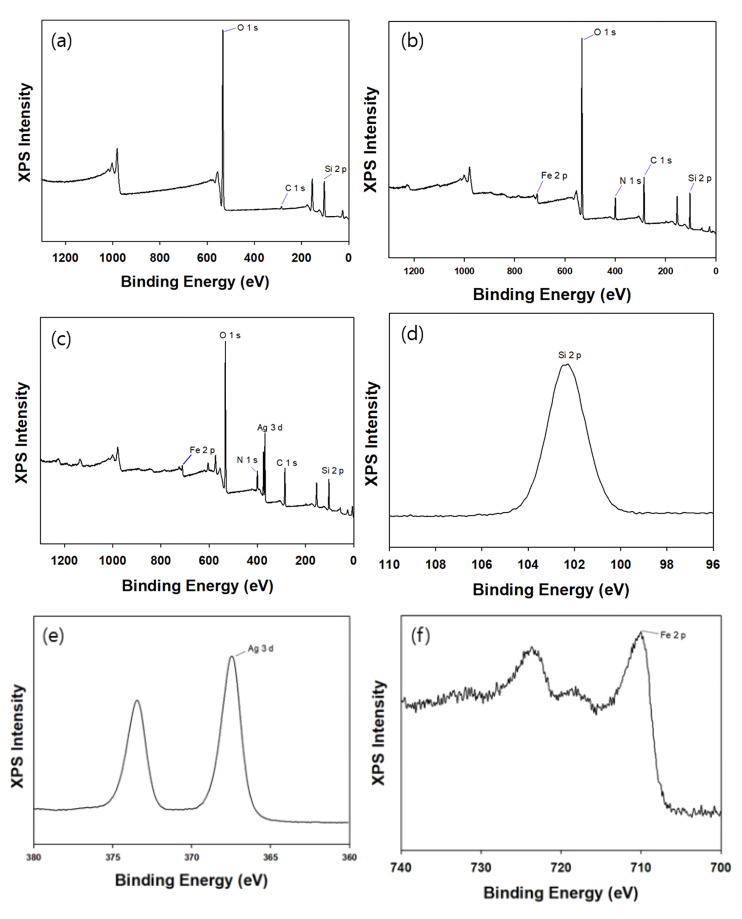
XPS spectra of (**a**) silica, (**b**) mag@silica, (**c**) mag@silica-Ag and (**d**) Si 2p spectrum, (**e**) Ag 3d spectrum. (**f**) Fe 2p spectrum of mag@silica-Ag.

XPS was employed to confirm that each step of the synthesis was successful and to examine the chemical composition of the materials. Figure 8a,b present the XPS spectra of silica and mag@silica, respectively. The presence of the Fe 2p peak in the mag@silica spectrum demonstrates, once again, that magnetite is present in the material. Besides the peaks due to the presence of Si, O, and Fe, the XPS spectrum of the mag@silica-Ag composite presents the peak corresponding to Ag. The high C content observed in the mag@silica and mag@silica-Ag might be due to the precursors used to synthesize silica (CTAB (C_19_H_42_BrN), AAPTMS (C_8_H_22_N_2_O_3_Si), and TEOS (C_8_H_2_O_4_Si)).

Table 3 presents the weight percent for each atom for mag@silica and mag@silica-Ag. As the silver was impregnated, the mass ratio of C, O, and Si decreased. Additionally, the Ag of mag@silica-Ag was 16.4%.

**Figure 9:**
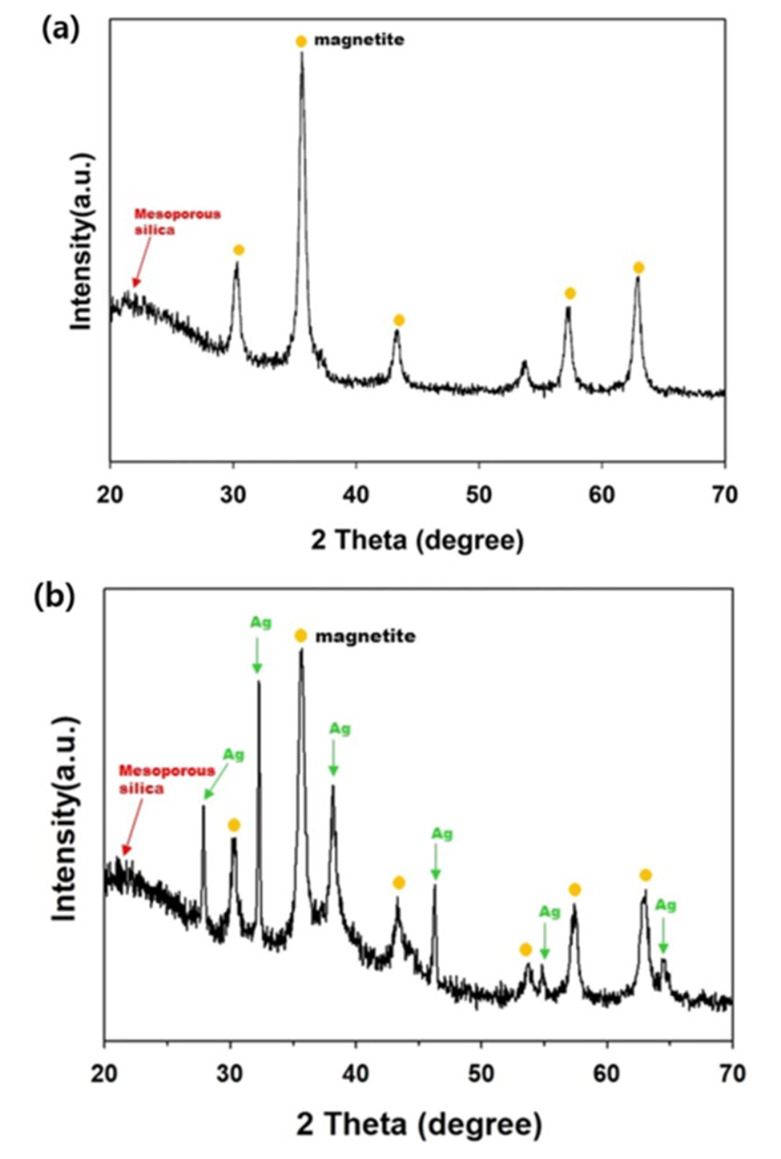
X-ray diffraction pattern of (**a**) mag@silica and (**b**) mag@silica-Ag. Yellow circle denotes the peaks corresponding to magnetite and the green arrows the peaks due to Ag.

Figure 9 displays the XRD patterns of the mag@silica and mag@silica-Ag composites. Both spectra present a peak at 2θ = 21.8° corresponding to the mesoporous silica. The peaks due to synthetic magnetite were identified from JCPDS (ref. code: 01-088-0315, Fe_3_O_4_) and were present in both samples [22,33,36]. Besides the peaks due to silica and magnetite, the mag@silica-Ag presented peaks at 2θ = 38.4117°, 2θ = 44.278°, and 2θ = 64.427° due to silver, identified from JCPDS (ref. code: 00-044-0783, Ag) [37].

**Figure 10:**
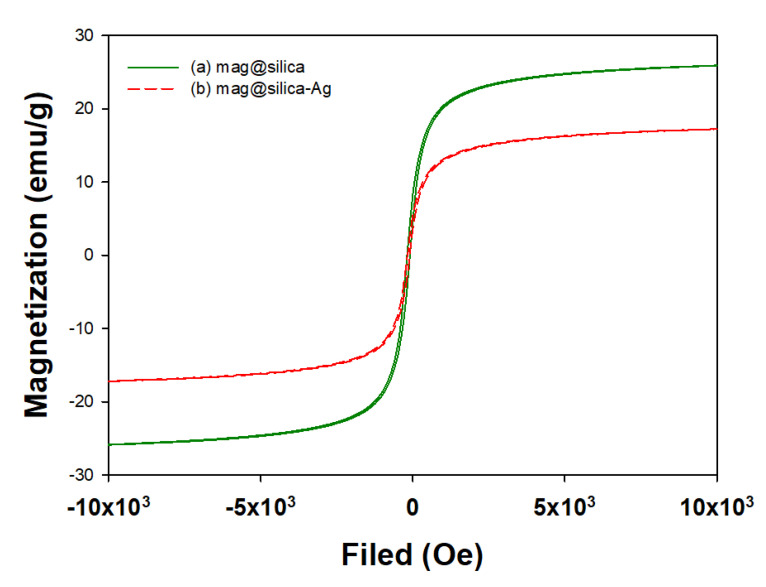
Magnetization curves for (**a**) mag@silica and (**b**) mag@silica-Ag.

The field-dependent magnetization curves of the mag@silica and mag@silica-Ag composites are shown in Figure 10. The magnetic properties of Fe_3_O_4_ samples were measured on the VSM. As shown in Figure 10, the magnetic saturation value for the mag@silica is about 26 emu/g and it decreases to 17 emu/g after the addition of silver. The gradual loss of magnetization strength can be attributed to the shielding effect of the silver layer. However, this effect was not observed on the magnetic separability of the nanoparticles from the bulk solution [38,39]. The saturation magnetization of the thiol-functionalized magnetic sawdust is 7.28 emu/g [37] while the one for magnetic polyoxometalates-based adsorbent is 8.19 emu/g [40]. Compared with the existing magnetic adsorbent values, the magnetite saturation value of mag@silica-Ag is high enough to be used for magnetic separation.

### 3.2. Adsorption Experiments

#### 3.2.1. Adsorption Isotherm

**Figure 11:**
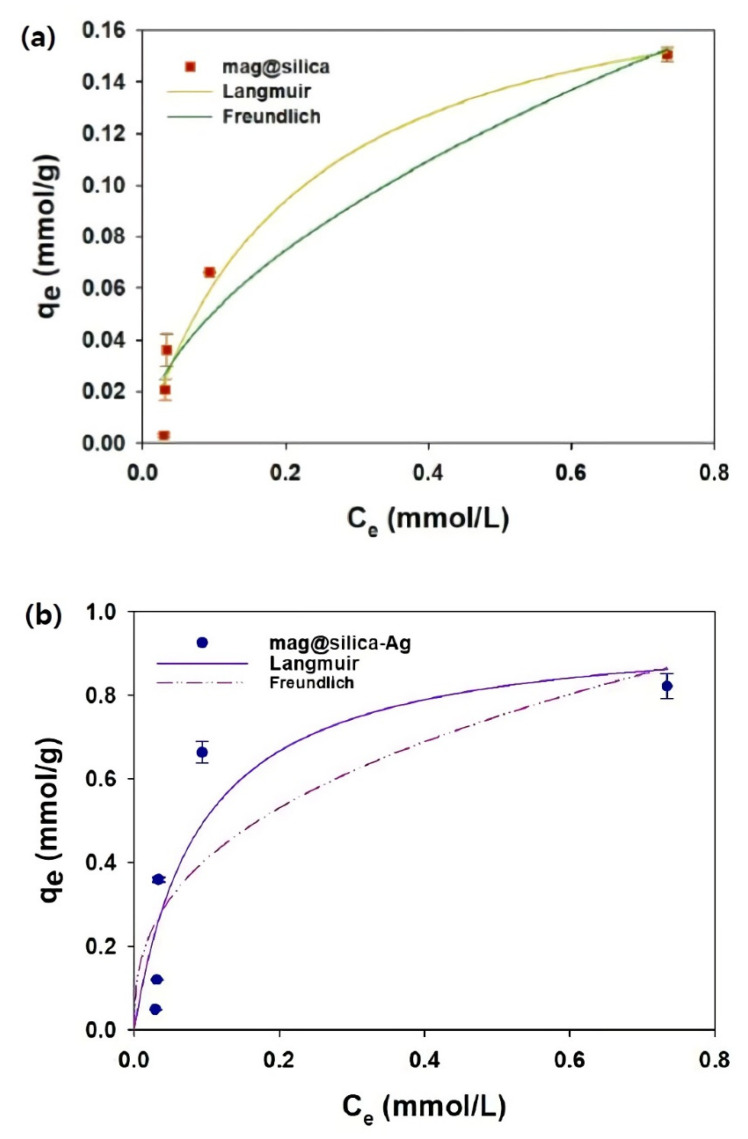
Sorption isotherm of I^−^ on (**a**) mag@silica and (**b**) mag@silica-Ag composite. Adsorbent 0.01 g, I^−^ solution 10 mL, 24 h, 25 °C.

The Langmuir and Freundlich sorption isotherms are presented in Figure 11, and the isotherm parameters obtained by fitting the experimentally observed sorption equilibrium data to the isotherm models are listed in Table 4. The amount of I^−^ adsorbed onto the synthetic sorbents increased with an increase in the initial I^−^ concentration, which demonstrates that the concentration gradient is the driving force of the sorption [41]. To test if silica has any effect on the adsorption of I^−^, bare silica was also used in the experiments, but no sorption was detected. The Langmuir model using mag@silica and mag@silica-Ag provides an adequate fit (R^2^ > 0.83) to the experimental data for I^−^ sorption onto the synthetic sorbents. The maximum sorption capacity (*q_m_*) of mag@silica-Ag was 0.82 mmol/g; this high adsorption is due to the presence of Ag. The good fitting of the experimental data to the Langmuir isotherm model, demonstrates that the I^−^ was adsorbed homogeneously onto the surface of the mag@silica-Ag composite via a monolayer sorption and that the silver was uniformly distributed throughout the surface. Moreover, the primary adsorption mechanism was determined to be a physicochemical adsorption [42].

The standard error of estimate (SEE) measures the variation of an observation made around the computed regression line. The SEE is the square root of sum of squared errors (SSE). The SSE formula and SEE formula are shown below.

(8) $$\mathrm{SSE}=\sum^{\;}{\left(y_{i}-{\hat{y}}_{i}\right)}^{2}$$

where $y_{i}$ is the predicted value and ${\hat{y}}_{i}$ is the actual value.

(9) $$\mathrm{SEE}=\sqrt{\frac{\mathrm{SSE}}{n-k-1}}$$

where *k* is the number of independent variables in the model, and *n* is the sample size. If SEE is zero, all the points fall on the regression line.

Table 5 lists the adsorption capacities of mag@silica-Ag and other adsorbents mentioned in the literature. Compared to the previously reported values, the material developed in the present study possesses a higher adsorption capacity and is expected to be suitable for large scale removal of iodide [31].

#### 3.2.2. Adsorption Kinetics

**Figure 12:**
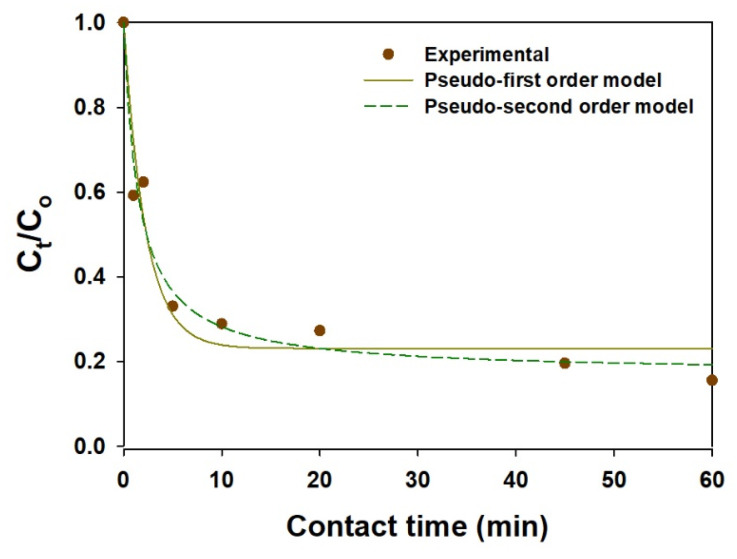
Kinetic adsorption of I^−^ on the mag@silica-Ag composite. Adsorbent 0.01 g, I^−^ solution 10 mL.

The adsorption kinetics play an important role when studying the efficiency and the cost for an actual application. In this context, the effect of contact time on the sorption of I^−^ on the mag@silica-Ag composite was examined. As shown in Figure 12, the mag@silica-Ag composite adsorbed I^−^ rapidly during the initial contact time (in 45 min, η = 80%) and reached adsorption equilibrium after 2 h. The sorption kinetics data were analyzed by PFOKM and PSOKM, and the predicted model parameters are listed in Table 6. Based on the regression coefficients (*R*^2^), the sorption kinetics data fit PSOKM better than PFOKM. Furthermore, the *q_m_* value calculated using PSOKM is in agreement with the adsorption isotherm results.

#### 3.2.3. Effect of pH

**Figure 13:**
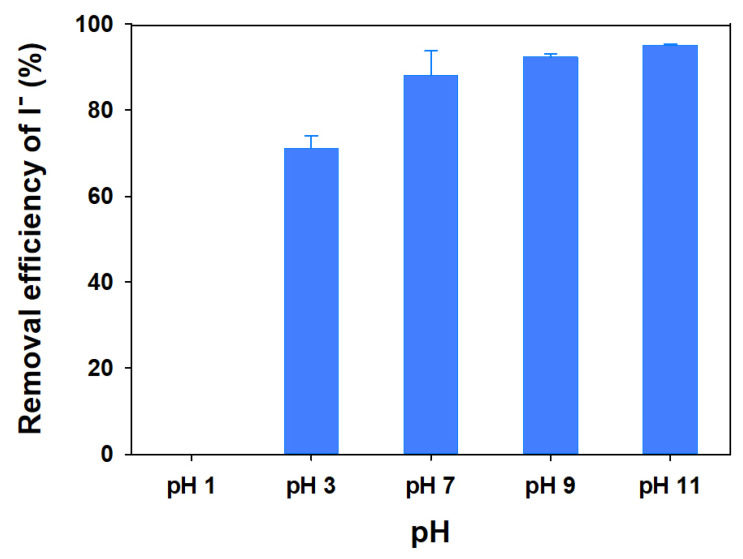
Removal efficiency of I^−^ as a function of pH on the mag@silica-Ag composite. Adsorbent 0.01 g, I^−^ solution 10 mL, 24 h, 25 °C.

The I^−^ sorption using mag@silica-Ag was studied at various pH values and it was found to be pH dependent, as can be seen from Figure 13. Iodide uptake occurs over the whole range of pH values studied, i.e., between 3 and 11. The uptake efficiency decreased as the pH decreases, as expected for an anion sorption isotherm, and in agreement with previous works [47]. The highest rate of iodide removal reached 95% at pH 11 while no removal was observed at pH 1.

As the pH decreases, the surface charge of the adsorbent decreases, thus it is expected for the adsorption capacity to decrease [48].

#### 3.2.4. Effect of Co-Existing Ions on I^−^ Sorption

**Figure 14:**
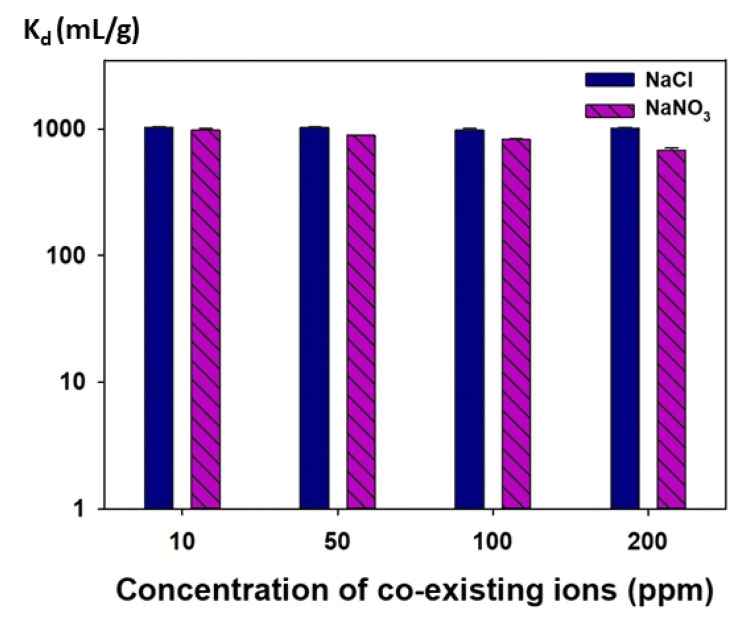
Distribution coefficients (*K_d_*) of I^−^ at different NaCl and NaNO_3_ concentrations on mag@silica-Ag composite.

The I^−^ uptake abilities of the mag@silica-Ag composite at different NaCl and NaNO_3_ concentrations expressed as distribution coefficient (*K_d_*) are shown in Figure 14. The distribution coefficient *(K_d_*) was determined using the following equation

(10) $$K_{d}=\frac{C_{o}-C_{e}}{C_{o}}\times \frac{V}{M}$$

where *C_o_* and *C_e_* represent the initial and equilibrium concentrations of I^−^, respectively. In addition, *V* denotes the volume of iodide solution (10 mL), and M is the mass of the adsorbent (mag@silica-Ag, 0.01 g). In the presence of 10–200 ppm NaCl or NaNO_3_, the *K_d_* values for I^−^ on mag@silica-Ag composite were about 10^3^. The NaCl and NaNO_3_ concentrations had a negligible influence on the *K_d_* values for I^−^ of the mag@silica-Ag. These high *K_d_* values indicate that this material can be successfully applicable for the selective removal of I^−^. At the same time, the adsorption rate of Cl^−^ and NO_3_^−^ was insignificant. The results demonstrate that, no matter the concentration of chloride and nitrite ions present in the wastewater, the material will selectively adsorb iodide ions.

## 4. Conclusions

In this study, mag@silica-Ag was synthesized for selective removal of I^−^, a by-product produced by the decomposition of uranium atoms in radioactive liquid waste. Silver was immobilized on the surface of mag@silica for the selective removal of I^−^, and the corresponding reaction process can be described as follows: $I^{-}+{\mathrm{Ag}}^{+}\to \mathrm{AgI}\left(s\right)$. The experimental results for the adsorption capacity of mag@silica-Ag, fit the Langmuir model perfectly, and the mag@silica-Ag composite was found to have a maximum adsorption capacity of 0.82 mmol/g. The Langmuir isotherm demonstrates that the I^−^ was adsorbed onto the homogeneous surface of mag@silica-Ag composite via a monolayer sorption. Besides the extreme good sorption capability, the material can be easily recovered due to the presence of magnetite. Last but not least, mag@silica-Ag proved to selectively remove iodide ions from solutions containing Cl^−^ and NO_3_^−^ alongside with I^−^. Therefore, mag@silica-Ag can be successfully used in the treatment of large-scale radioactive liquid wastewaters.

## Figures and Tables

**Table 1:** Component ratio of mag@silica-Ag by EDS.

Element	Wt (%)	Atomic (%)
N	5.84	11.10
O	34.30	57.07
Si	13.39	12.69
Fe	33.43	15.93
Ag	13.04	3.22
Total	100	100

**Table 2:** Physical properties of the silica, mag@silica, and mag@silica-Ag.

Samples	BET Surface Area (m^2^g^−1^)	Pore Size (nm)	Pore Volume (cm^3^g^−1^)
silica	768	5	0.7
mag@silica	128	4	0.4
mag@silica-Ag	97	4	0.3

**Table 3:** The elemental ratio (Wt %) of mag@silica and mag@silica-Ag from XPS results.

Samples	C	O	Si	Fe	Ag
mag@silica	21.2	41.0	32.1	5.7	
mag@silica-Ag	16.8	35.0	25.1	6.7	16.4

**Table 4:** Langmuir and Freundlich isotherm parameters for the adsorption of I^−^ onto mag@silica and mag@silica-Ag.

	Langmuir Model				Freundlich Model			
	*q_m_* (mmol/g)	*b* (L/mmol)	*R* ^2^	SEE	*K_f_*	*N*	*R* ^2^	SEE
mag@silica	0.13	7.57	0.83	0.1511	0.9735	0.38	0.76	0.1893
mag@silica-Ag	0.82	11.01	0.84	0.0260	0.1471	0.42	0.72	0.0337

**Table 5:** Comparison of maximum adsorption capacity with previously developed materials and mag@silica-Ag.

Adsorbent	Maximum Adsorption Capacity (*q_max_*) (mmol/g)	Reference
mag@silica-Ag	0.82	This study
Cu/Cu_2_O hybrids	0.18	[32,43]
Cu_2_O/Cu-C	0.32	[44]
Mg-Al LDO/SiO_2_	0.55	[45]
LDH	0.41	[46]
T3NT	0.5	[3]
T3NL	0.2	
T3NF	0.1	
Silver-impregnated activated carbon	0.097	[21,44]
Electric Arc Furnace Slag	0.34	[4]
Nanocomposite membranes	0.012	[31]

**Table 6:** Kinetic adsorption parameters obtained using PFOKM and PSOKM on the mag@silica-Ag composite at 50 ppm of I^−^.

Concentration of I^−^ (mg/L)	Kinetic Model	Parameters		
50	PFOKM	*q_e_* (mg/g) 38.4637	k_1_ (min^−1^) 0.4518	R^2^ 0.8319
	PSOKM	*q_e_* (mg/g) 41.3988	k_2_ (g/mg/min) 0.0158	R^2^ 0.9048

## Data Availability

The data presented in this study are available on request from the corresponding author. The data are not publicly available due to institutional and national data sharing restrictions.
